# Superior humoral immunity in vaccinated SARS-CoV-2 convalescence as compared to SARS-COV-2 infection or vaccination

**DOI:** 10.3389/fimmu.2022.1031254

**Published:** 2022-10-31

**Authors:** Krystallenia Paniskaki, Margarethe J. Konik, Moritz Anft, Toni L. Meister, Corinna Marheinecke, Stephanie Pfaender, Jasmin Jäger, Adalbert Krawczyk, Markus Zettler, Sebastian Dolff, Timm H. Westhoff, Hana Rohn, Ulrik Stervbo, Oliver Witzke, Nina Babel

**Affiliations:** ^1^ Department of Infectious Diseases, West German Centre of Infectious Diseases, University Hospital Essen, University Duisburg-Essen, Essen, Germany; ^2^ Center for Translational Medicine and Immune Diagnostics Laboratory, Medical Department I, Marien Hospital Herne, University Hospital of the Ruhr-University Bochum, Bochum, Germany; ^3^ Department of Molecular and Medical Virology, Ruhr-University Bochum, Bochum, Germany; ^4^ Medical Department I, Marien Hospital Herne, University Hospital of the Ruhr-University Bochum, Herne, Germany; ^5^ Berlin Institute of Health at Charité – University Clinic Berlin, BIH Center for Regenerative Therapies (BCRT) Berlin, Berlin, Germany

**Keywords:** SARS-CoV-2, T cells, neutralizing antibodies, vaccine, adaptive immunity

## Abstract

Emerging variants of concern (VOC) raise obstacles in shaping vaccination strategies and ending the pandemic. Vaccinated SARS-CoV-2 convalescence shapes the current immune dynamics. We analyzed the SARS-CoV-2 VOC-specific cellular and humoral response of 57 adults: 42 convalescent mRNA vaccinated patients (C+V+), 8 uninfected mRNA vaccinated (C-V+) and 7 unvaccinated convalescent individuals (C+V-). While C+V+ demonstrated a superior humoral SARS-CoV-2 response against all analyzed VOC (alpha, delta, omicron) compared to C-V+ and C+V-, SARS-CoV-2 reactive CD4+ and CD8+ T cells, which can cross-recognize the alpha, delta and omicron VOC after infection and/or vaccination were observed in all there groups without significant differences between the groups. We observed a preserved cross-reactive C+V+ and C-V+ T cell memory. An inferior humoral response but preserved cross-reactive T cell memory in C+V- compared to C+V+ was observed, as well as an inferior humoral response but preserved cross-reactive T cell memory in C+V- compared to C-V+. Adaptive immunity generated after SARS-CoV-2 infection and vaccination leads to superior humoral immune response against VOC compared to isolated infection or vaccination. Despite the apparent loss of neutralization potential caused by viral evolution, a preserved SARS-CoV-2 reactive T cell response with a robust potential for cross-recognition of the alpha, delta and omicron VOC was detected in all studied cohorts. Our results may have implications on current vaccination strategies.

## 1 Introduction

The emergence of numerous VOC since the late 2020 sustains the COVID-19 pandemic and challenges our immunological and virological understanding of the SARS-CoV-2 virus, both being crucial for shaping health policies and vaccination strategies. Waning of neutralizing antibodies (NAb) 3-4 months after vaccination or infection has been documented, challenging the achievement of sterilizing immunity (1, 2). On the other hand, it is hypothesized, that preserved SARS-CoV-2 reactive T cells might prevent severe COVID-19 disease in case of reinfection or vaccine breakthrough infection and therefore reduce hospitalizations (3–7).

Independent studies demonstrate that spike (S) mutations can lead to loss of T cell recognition or presentation of some epitopes restricted by common HLA alleles enhancing potentially immune escape (8–10). However, Grifoni et al. and Choi et al. conclude that a large breadth of epitopes are recognized in human populations, making it unlikely that SARS-CoV-2 variants might escape T cell recognition at the population level (11, 12).

SARS-CoV-2 immune dynamics are shaped by the immune status of different subpopulations depending on their contact with SARS-CoV-2 antigens; a) vaccinated uninfected, b) vaccinated infected, c) convalescent unvaccinated, d) uninfected unvaccinated and finally e) immunocompromised individuals. Emerging data begin to draw the cellular and humoral responses against VOC in unvaccinated convalescence, vaccinated convalescence, or vaccinated adults separately (13–19). However, data comparing VOC cross-recognition and SARS-CoV-2 memory among vaccinated convalescent and uninfected vaccinated adults is currently missing. To address the protective capacity against the most infuencial VOC and provide insights in the evolution of protective immunity in context of SARS-CoV-2 mutation, we performed cellular and humoral immune profiling reactive against the most influential VOC (alpha, delta and omicron) in 42 mRNA vaccinated convalescent adults, 8 uninfected mRNA vaccinated and 7 unvaccinated convalescent individuals.

## 2 Materials and methods

### 2.1 Study participants

We used peripheral blood mononuclear cells (PBMCs) and serological samples from 42 vaccinated convalescent COVID-19 individuals (further referred as C+V+), 8 vaccinated uninfected with SARS-CoV-2 adults (further referred as C-V+) and 7 unvaccinated convalescent (further referred as C+V-) individuals.

As we studied convalescent subjects, a direct molecular sequencing of the viral variant responsible for SARS-CoV-2 infection was not possible. Therefore, we determined the viral variants that the study participants were infected with, relying on temporal viral spreading defined by epidemiologcal trends in Germany. The SARS-CoV-2 diagnosis for the C+V+ and C+V- participants was set between June 2020 and May 2021. According to epidemiological data of the Robert Koch Institute alpha variant drove and overrepresented SARS-CoV-2 infections in Germany for the first half of 2021 (https://www.rki.de/DE/Content/InfAZ/N/Neuartiges_Coronavirus/Virologische_Basisdaten.html;jsessionid=93905B429A0980A796F15E1AA070EF2C.internet102?nn=13490888#doc14716546bodyText14).

The study was approved by the Ethics Committee of the University Hospital Essen (20-9753-BO). A part of the patient data was included in the research article "Increased migratory/activated CD8+ T cell and low avidity SARS-CoV-2 reactive cellular response in post-acute COVID-19 syndrome (PASC)" currently under consideration for publication. Written informed consent was obtained from all participants. Demographic and clinical characteristics are provided in **Table 1**.

**Table 1:** Demographic and clinical characteristics of the study cohorts.

	C+V+ (N=42)	C-V+(N=8)	C+V- (N=7)
**Age years -median (range)**	48.5 (19-68)	30 (22-46)	46 (20-65)
**Female gender N (%)**	29/13 (69/31)	7/1 (88/12)	5/2 (71/29)
Time since COVID-19 Diagnosis (months)*	11 (7-16)	0 (0)	9 (5-12)
**COVID-19 Severity N (%)**			
*Moderate*	39(93)	0 (0)	7 (100)
*Severe*	2 (5)	0 (0)	0 (0)
*Critical*	1 (2)	0 (0)	0 (0)
Time last vaccination up to recruitment (months)**	4 (1-8)	5 (2-5)	N/A
**Number of antigenic contacts (range)**	2 (2-4)	3	1
**Number of mRNA vaccinations**			
*1 N (%)*	28 (67)	0 (0)	0 (0)
*2 N (%)*	12 (29)	0 (0)	0 (0)
*3 N (%)*	2 (4)	8 (100)	0 (0)
**VOC**			
*WT N(%)*	30 (71)	0 (0)	4 (57)
*Alpha N(%)*	12 (29)	0 (0)	3 (43)
*Delta N(%)*	0 (0)	0 (0)	0 (0)
*Omicron N(%)*	0 (0)	0 (0)	0 (0)

*Months from SARS-CoV-2 Diagnosis (positive PCR) till the time point of study recruitment.

**Months from last vaccination up to study recruitment

### 2.2 Preparation of PBMCs

As previously described (20), peripheral blood was collected in S-Monovette K3 EDTA blood collection tubes (Sarstedt). Collected blood was prediluted in PBS/BSA (Gibco) at a 1:1 ratio and underlaid with 15 mL of Ficoll-Paque Plus (GE Healthcare). Tubes were centrifuged at 800g for 20 min at room temperature. Isolated PBMCs were washed twice with PBS/BSA and stored at -80°C until use. The cryopreserved PBMCs were thawed by incubating cryovials 2-3 minutes at 37°C in bead bath, washed twice in 37°C RPMI 1640 media (Life Technologies) supplemented with 1% penicillin-streptomycin-glutamine (Sigma-Aldrich), and 10% fetal calf serum (PAN-Biotech) medium, and incubated overnight at 37°C.

### 2.3 Flow cytometry - measurement of SARS-CoV-2 reactive T cells

As previously described, PBMCs were plated in 96-U-Well plates in RPMI 1640 media (Life Technologies) (21, 22). Each well was stimulated with one of the following SARS-CoV-2 proteins: the pool of B1.617.2 (delta) S mutant peptides (Miltenyi Biotec), their corresponding position in wildtype (WT) pool of peptides (Dref) (Miltenyi Biotec), the pool of B.1.1529 (omicron) S mutant peptides (Miltenyi Biotec), their corresponding position in WT pool of peptides (Oref) (Miltenyi Biotec) and the complete sequence of B.1.1.7 D614G S mutant (JPT Peptide Technologies) or WT S-protein (Miltenyi Biotec) or left untreated as a control for 16 h. The proteins were dissolved per manufacturer’s directions. As a positive control, cells were stimulated with staphylococcal enterotoxin B (1 μg/mL, Sigma-Aldrich). After 2 h, brefeldin A (1 μg/mL, Sigma-Aldrich) was added. Detailed listing of the antibody panels for general phenotyping and T cell activation *ex vivo* is in **Table S1**. The PBMCs stimulated overnight were stained with optimal concentrations of antibodies for 10 min at room temperature in the dark. Stained cells were washed twice with PBS/BSA before preparation for intracellular staining using the Intracellular Fixation & Permeabilization Buffer Set (Thermo Fisher Scientific) as per the manufacturer’s instructions. Fixed and permeabilized cells were stained for 30 min at room temperature in the dark with an optimal dilution of antibodies against the intracellular antigen. All samples were immediately acquired on a CytoFLEX flow cytometer (Beckman Coulter). Quality control was performed daily using the recommended CytoFLEX daily QC fluorospheres (Beckman Coulter). No modification to the compensation matrices was required throughout the study. Antigen-reactive responses were considered positive after the non-reactive background was subtracted, and more than 0.01% were detectable. Negative values were set to zero.

### 2.4 SARS-CoV-2 N IgG and neutralization assay

Human IgG autoantibodies against the SARS-CoV-2 nucleocapsid (N) protein were detected using commercial ELISA kits (EUROIMMUN) according to the manufacturer’s instructions.

As previously described (22), for the virus neutralization assay, sera were incubated for 30 min at 56°C in order to inactivate complement factors. Single cycle VSV∗ΔG(FLuc) pseudoviruses bearing the SARS-CoV-2 WT S (D614G) protein or SARS-CoV-2 alpha S protein, or SARS-CoV-2 delta or SARS-CoV-2 omicron S protein virus in the envelope were incubated with quadruplicates of two-fold serial dilutions of immune sera in 96-well plates prior to infection of Vero E6 cells (1x104 cells/well) in DMEM + 10% FBS (Life Technologies). At 18 hours post infection, firefly luciferase (FLuc) reporter activity was determined, based on CPE values, the 50% neutralization capacity (ND50 value) was calculated using non-linear regression.

### 2.5 Statistics

Flow cytometry data were analyzed using FlowJo version 10.6.2 (BD Biosciences); gating strategies are presented in **Figure S1**. For the analysis of anti-SARS-CoV-2 reactive T cells, a threshold of 0.01% was employed to define a detectable response. Single stains and fluorescence-minus-one controls were used for gating. Gates of each study participant were adjusted according to the negative control. CD4+ T cells expressing CD154 and CD137 and CD8+ T cells expressing CD137 were defined as reactive T cells. Statistical analysis was performed using GraphPad Prism v7. Categorical variables are summarized as numbers and frequencies; quantitative variables are reported as median and interquartile range. Normality tests were performed with the Shapiro-Wilk Test. All applied statistical tests are two-sided. Kruskal-Wallis Test and Mann-Whitney-Test were applied to perform comparisons. Spearman´s rank correlation coefficient was used to explore a possible correlational relationship between T cells and neutralizing antibodies. The age between the two cohorts was compared using unpaired two-tailed t-test, and gender was compared using two-tailed Fisher’s exact test. p values below 0.05 were considered significant; only significant p values are reported in the figures. p values were not corrected for multiple testing, as this study was of an exploratory nature.

## 3 Results

### 3.1 Characterization of the study groups

Our study group comprised 42 vaccinated convalescent COVID-19 individuals (further referred as C+V+), 8 vaccinated individuals without previous COVID-19 (further referred as C-V+) and 7 unvaccinated COVID-19 convalescent (further referred as C+V-) individuals. All study participants had a negative SARS-CoV-2 nasal swab tested *via* RT-PCR on recruitment. The C-V+ study group was seronegative for N-SARS-CoV-2 IgG, excluding an asymptomatic SARS-CoV-2 infection in the past. Most individuals 93% (n=39) experienced moderate COVID-19 during the acute phase of COVID-19 disease without need for hospitalization, whereas only 7% (n=3) were severely or critically ill and hospitalized.

67% (n=28) of the C+V+ individuals received one anti-SARS-CoV-2 mRNA vaccination after the SARS-CoV-2 infection, 29% (n=12) 2 vaccinations and 4% (n=2) 3 vaccinations. All C-V+ individuals received 3 anti-SARS-CoV-2 mRNA vaccinations.

The median time since COVID-19 convalescence for the C+V+ study group was 11 months (range 7-16 months). The median time since the last vaccination was 4 and 5 months for the C+V+ and C-V+ study group, respectively (range 1-8 & 2-5 months respectively). 71% (n=30) of the C+V+ study group was infected with the WT whereas 29% (n=12) with the alpha variant.

The median age of the C+V+ study group was 48.5 years (range 19-68 years), whereas the control cohort was significantly younger, with a median age of 30 years (range 22-46 years, p=0.003 two tailed unpaired t test). The C+V+ and C-V+ cohorts comprised of 69% (n=29) and 88% (n=7) female participants, respectively and showed no significant gender difference (Fisher´s exact test, p<0.05). The demographic and clinical characteristics of the study cohorts are presented in **Table 1**.

### 3.2 Superior humoral SARS-CoV-2 response among C+V+ against all VOC compared to C-V+, accompanied by sustained cellular immunity with comparable magnitude among C+V+ and C-V+

Up to date, humoral immunity and more concrete high titers of neutralizing antibodies are considered the pylons of protection against SARS-CoV-2 infection (1). Therefore, we performed a pseudovirus neutralization assay against WT, alpha, delta and omicron VOC. First, we compared the neutralization capacity of WT, alpha, delta and omicron VOC in C+V+ cohort. WT NAb titers were significantly higher compared to the alpha, delta and omicron VOC (**Figure S2A**) (Kruskal-Wallis test, p<0.0001). Furthermore, we observed a steady decline of NAb titers within the known SARS-CoV-2 variant evolution from WT to the most current VOC omicron. To quantitatively assess the loss of neutralizing capacity following the viral evolution, we normalized the median NAb of VOCs to WT: alpha/WT 72% (range 0-352%); delta/WT 54% (range 0-237); omicron/WT 31% (range 0-100%). Especially for the most current VOC omicron, this calculation demonstrates an approximately 70% loss of neutralizing potential among the C+V+ cohort (**Figure S2A**).

A similar pattern of humoral immunity was found for the C-V+ cohort(**Figure S2B**). A comparable loss of neutralizing capacity was also demonstrated for the C-V+ group: alpha/WT 84% (range 29-148%), delta/WT 43% (range 28-43), omicron/WT 21% (range 0-89%). All in all, we observed a steady decline of NAb titers across viral evolution with the lowest neutralization capacity against omicron VOC.

**Figure 1:**
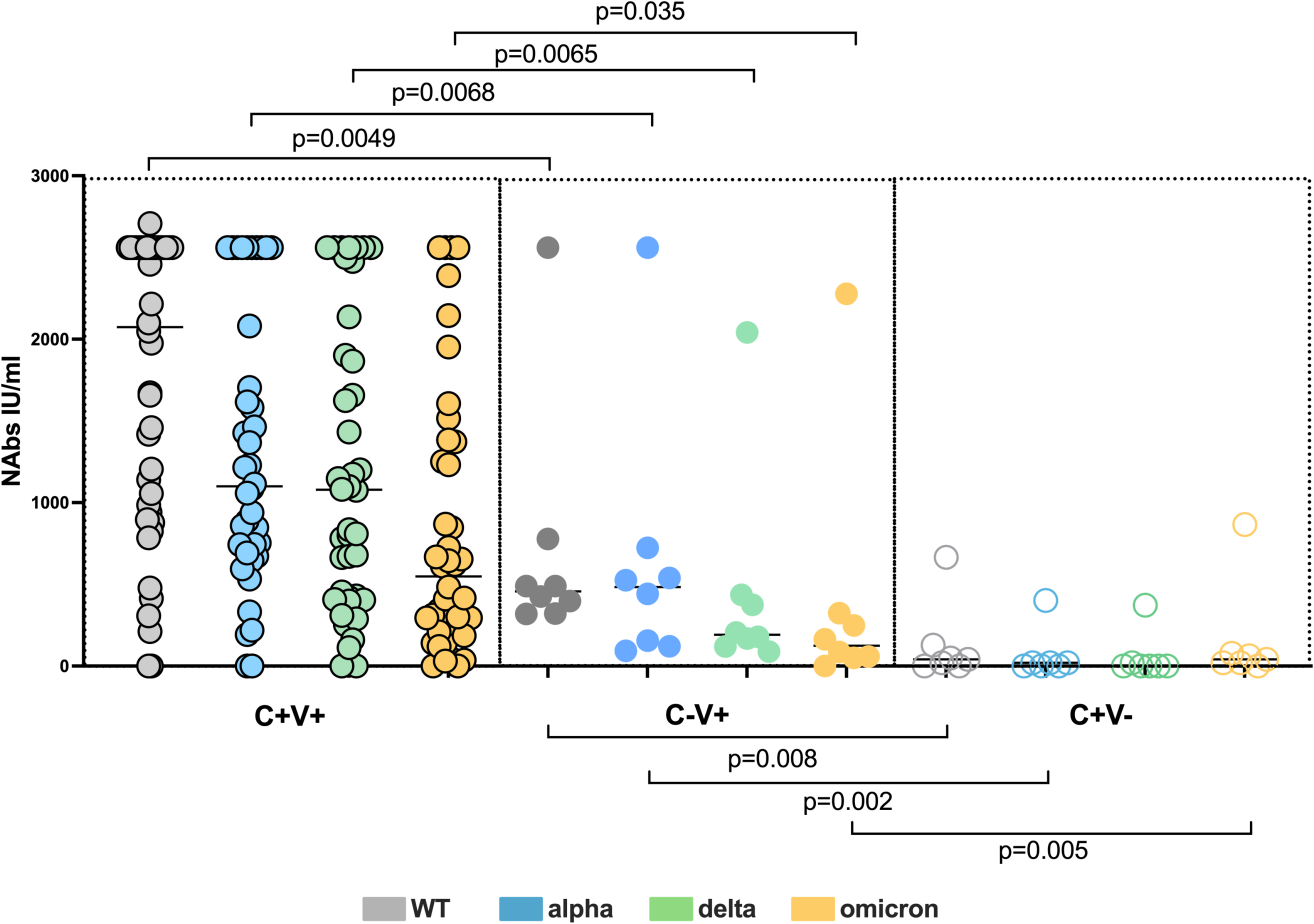
Superior humoral SARS-CoV-2 response among C+V+ and C-V+ against all VOC compared to C-V+. Comparison of WT, alpha, delta and omicron NAb titers among C+V+, C-V+ and C+V- study subjects. Scatterplots show line at median. Unpaired data were compared with Mann-Whitney-test. The assay detection limit for ND50 is 20. P<0.05 was considered significant, only significant p values are documented in the figures. To simplify the figure and the data presentation the significant results among C+V+ and C+V- are not documented in the figure (Results section 3.3).

Next, we compared the neutralizing capacity between the C-V+ und and C+V+ study groups. The C+V+ cohort possessed significantly higher titers of neutralizing antibodies compared to the C-V+ cohort against all VOC (Mann Whitney test, WT NAb C+V+ vs C-V+ p=0.0049) (Mann Whitney test, alpha NAb C+V+ vs C-V+ p=0.0068) (Mann Whitney test, delta NAb C+V+ vs C-V+ p=0.0065) (Mann Whitney test, omicron NAb C+V+ vs C-V+ p=0.035) (**Figure 1**). However, the absolute loss of neutralizing capacity among the C+V+ and C-V+ cohorts did not show any significant differences (Fisher´s exact test, C+V+ vs C-V+ omicron/WT ratio p=0.1464) (Fisher´s exact test, C+V+ vs C-V+ delta/WT ratio p=0.1569) (Fisher´s exact test, C+V+ vs C-V+ alpha/WT ratio p=0.0597) among the two cohorts (**Figure 1**). These results indicate that the immunocompetent vaccinated convalescent adults generate higher NAb titers compared to immunocompetent uninfected vaccinated adults, however the loss of neutralization potential is proportionally equal.

**Figure 2:**
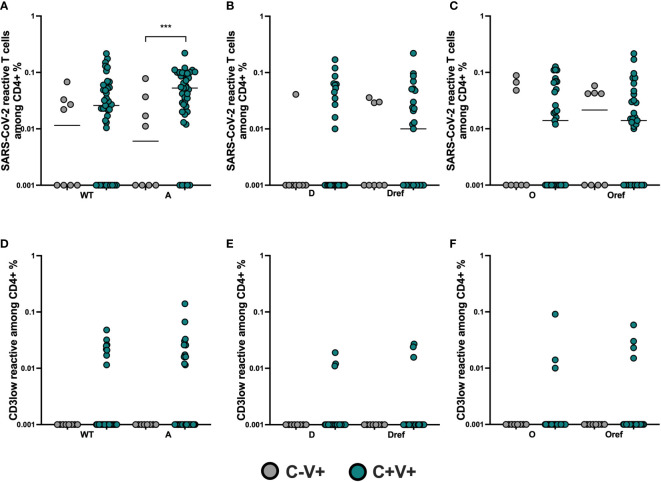
Comparable magnitude of SARS-CoV-2 CD4+ T cell response in C+V+ and C-V+. Comparison of SARS-CoV-2 S-reactive T cells among C+V+ and C-V+ study subjects. **(A)** Frequencies of WT- and alpha **(A)**-reactive CD4+ T cells. **(B)** Frequencies of delta (D)- and delta reference pool (Dref)-reactive CD4+ T cells. **(C)** Frequencies of omicron (O)- and omicon reference pool (Oref)-reactive CD4+ T cells. **(D)** Avidity of SARS-CoV-2 reactive CD4+ T cells was approached by determining the CD3low+ cells among CD4+CD154+CD137+ cells. Frequencies of WT- and alpha-reactive CD4+CD3low+ T cells. **(E)** Frequencies of delta- and Dref-reactive CD4+CD3low+ T cells. **(F)** Frequencies of omicron- and Oref-reactive CD4+CD3low+ T cells. Antigen-reactive responses were considered positive after the non-reactive background was subtracted, and more than 0.01% were detectable. Scatterplots show line at median. Unpaired data were compared with Mann-Whitney-test. P<0.05 was considered significant, only significant p values are documented in the figures. ***p<0.001.

**Figure 3:**
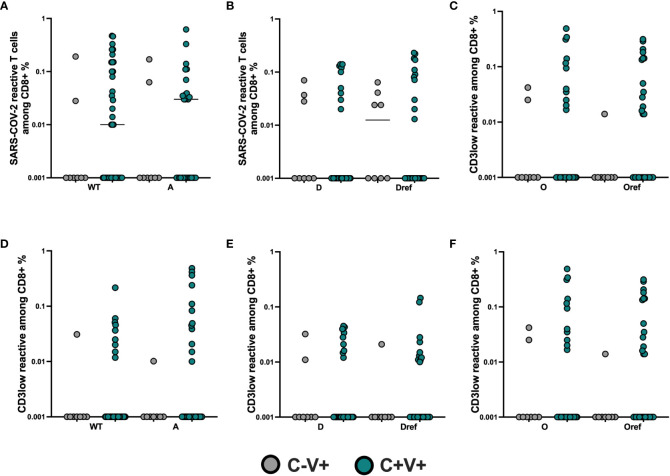
Comparable magnitude of SARS-CoV-2 CD8+ T cell response in C+V+ and C-V+. Comparison of SARS-CoV-2 S-reactive T cells among C+V+ and C-V+ study subjects. **(A)** Frequencies of WT- and alpha-reactive CD8+ T cells. **(B)** Frequencies of delta- and Dref-reactive CD8+ T cells. **(C)** Avidity of SARS-CoV-2 reactive CD8+ T cells was approached by determining the CD3low+ cells among CD8+CD137+ cells. Frequencies of omicron- and Oref-reactive CD8+ T cells. **(D)** Frequencies of WT- and alpha-reactive CD8+CD3low+ T cells. **(E)** Frequencies of delta- and Dref-reactive CD8+CD3low+ T cells. **(F)** Frequencies of omicron- and Oref-reactive CD8+CD3low+ T cells. Antigen-reactive responses were considered positive after the non-reactive background was subtracted, and more than 0.01% were detectable. Scatterplots show line at median. Unpaired data were compared with Mann-Whitney-test. P<0.05 was considered significant, only significant p values are documented in the figures.

At last, we compared the S-reactive T cell responses among the C+V+ and C-V+ study groups for all analyzed VOCs. We stimulated overnight PBMCs with the pool of delta S mutant peptides, their corresponding position in WT pool of peptides (Dref), the pool of omicron S mutant peptides, their corresponding position in WT pool of peptides (Oref), the complete sequence of alpha S mutant or WT S-protein. First, we analyzed the frequencies of SARS-CoV-2 reactive CD4+ T cells. We found similar frequencies of WT, delta and omicron reactive CD4+ T cells(**Figures 2A–C**) with comparable avidity (**Figures 2D–F**), however the alpha reactive CD4 T cells showed higher frequencies among the C+V+ group (**Figure 2A**) (Mann-Whitney and Fisher´s exact test). The frequencies of SARS-CoV-2 reactive CD8+ T cells were similar against all tested peptides (**Figures 3A–C**), as well as their avidity(**Figures 3D–F**).

### 3.3 Inferior humoral response but preserved SARS-CoV-2 reactive T cell response in C+V- compared to C+V+ and C-V+

To be able to integrate our findings into the current immune dynamics and to avoid one sided interpretation of our results, we additionally analyzed the humoral and cellular SARS-CoV-2 response of 7 unvaccinated convalescent COVID-19 adults with a median convalescent time of 9 months (range 5-12 months), further referred as C+V-. We found significantly higher titers of NAb against all studied VOC among the C+V+ individuals compared to the C+V- (Mann Whitney test, WT NAb C+V+ vs C+V- p<0.0001) (Mann Whitney test, alpha NAb C+V+ vs C+V- p<0.0001) (Mann Whitney test, delta NAb C+V+ vs C+V- p<0.0001) (Mann Whitney test, omicron NAb C+V+ vs C+V- p=0.0028) (**Figure 1**). Similar data were observed comparing C+V- to C-V+ group (**Figure 1**).

**Figure 4:**
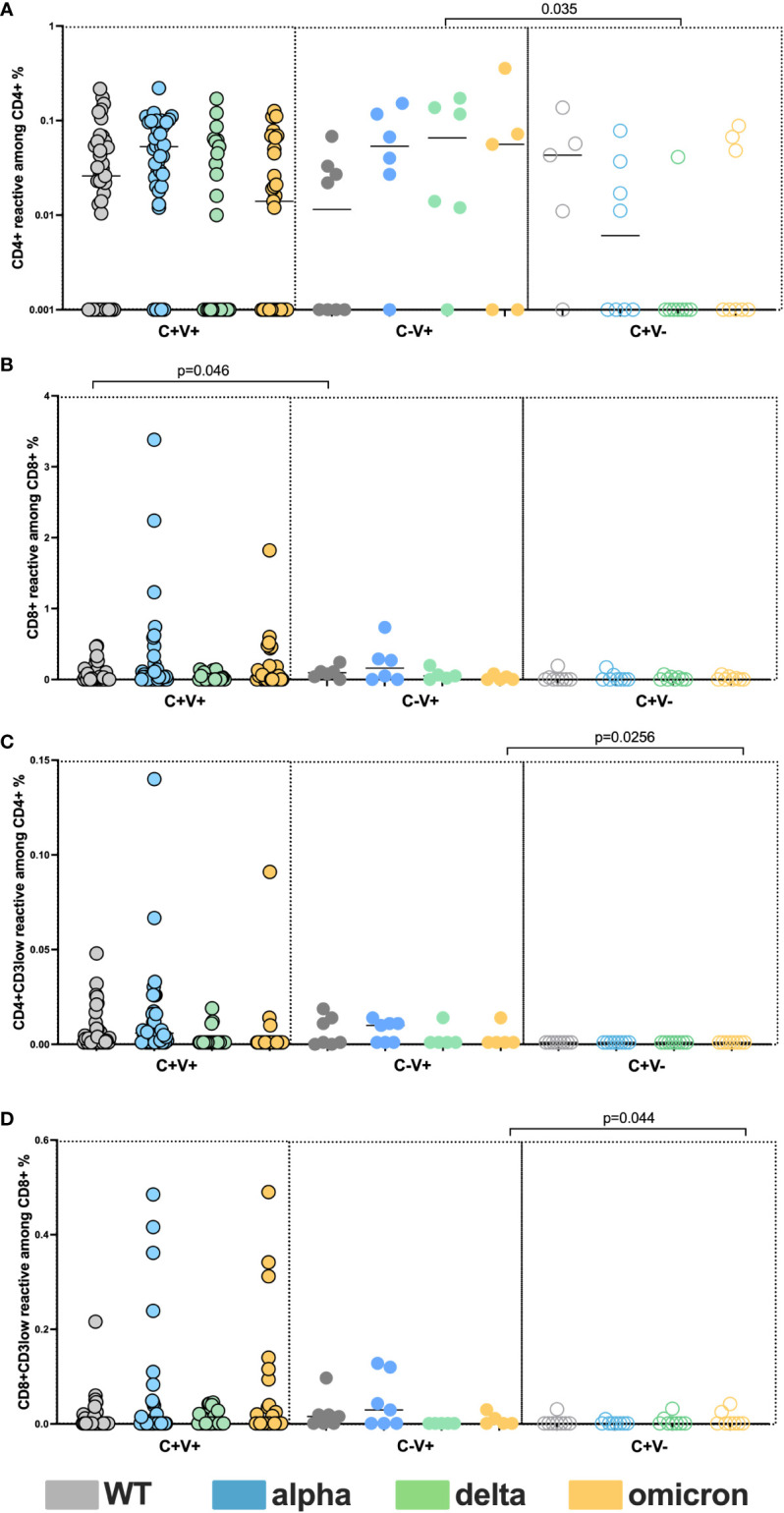
Preserved cross-reactive T cell memory among C+V+, C-V+ and C+V-. Comparison of WT, alpha, delta and omicron-reactive T cells among C+V+, C-V+ and C+V. **(A)** Frequencies of SARS-CoV-2 reactive CD4+ T cells. **(B)** Frequencies of SARS-CoV-2 reactive CD8+ T cells. **(C)** Frequencies of SARS-CoV-2 reactive CD4+CD3low+ T cells. **(D)** Frequencies of SARS-CoV-2 reactive CD8+CD3low+ T cells. Scatterplots show line at median. Unpaired data were compared with Mann-Whitney-test.

Next, we analyzed the SARS-CoV-2 reactive cellular response among the C+V+ and C+V-. We detected WT, alpha, delta and omicron reactive CD4+ and CD8+ T cells with similar frequencies between the two cohorts(**Figures 4A, B**). The CD3low analysis showed similar functional avidity among the cohorts (**Figures 4C, D**). These results demonstrate a superior humoral response among the C+V+ adults compared to C+V-, however similar cellular response with comparable cross-recognition potential against the studied VOC. Comparing C+V- group to C-V+ demonstrated similar results (**Figure 4**).

### 3.4 SARS-CoV-2 reactive CD4+ and CD8+ T cells cross-recognize VOC after infection and/or vaccination

To address the interesting question of VOC cross-recognition inside each study group separately we performed a reanalysis of our data. We demonstrate similar frequencies of WT, alpha, delta and omicron CD4+ & CD8+ reactive T cells in C+V+ individuals (Mann Whitney test, p<0.05) (**Figures S3A, C**). Furthermore, we performed an analysis of the CD3low subset among reactive CD4+ and CD8+ T cells (gating strategy, **Figure S1**) to address their avidity against the different variants (22, 23). We detected similar frequencies of reactive CD4+ and CD8+CD3low T cells against all studied VOC, except for the alpha variant where alpha CD4+CD3low reactive T cells showed significantly higher frequencies compared to WT CD4+CD3low reactive T cells (Mann Whitney test, p=0.022) (**Figures S3B, D**). Our results demonstrate that SARS-CoV-2 reactive CD4+ and CD8+ T cells recognize alpha, delta and omicron VOC 11 months after infection in vaccinated adults with equal avidity.

As next we assessed the reactive T cell response among the C-V+ study group. In the same line with the C+V+ group, we detected similar frequencies of WT, alpha, delta and omicron reactive CD4+ and CD8+ T cells (**Figures S3E, G**). The frequencies of CD3low reactive T cells showed no statistical difference (**Figures S3F, H**).

### 3.5 Preservation of functional activity in T-cells directed against different VOC

In addition to the quantification, avidity and the phenotypic differentiation analysis, we aimed for the analysis of the functional activity as defined by cytokine production. We found similar frequencies of monofunctional IL2, IFNg, TNFa or GrB producing WT, alpha, delta and omicron reactive CD4+ and CD8+ T cells (**Figures S4, S5**). Our results suggest that SARS-CoV-2 specific T cells preserve their functionality against the studied VOC.

### 3.6 Preserved cross-reactive C+V+ and C-V+ T cell memory & predominance of the T_CM_ CD4+ cell memory among the C+V+

To evaluate the SARS-CoV-2 T cell memory in details, we analyzed differentiation phenotype using the markers CCR7 and CD45RA (T_CM_=CD45RA-CCR7+, T_NAIVE_=CD45RA+CCR7+, T_EM_=CD45RA-CCR7- T_EMRA_=CD45RA+CCR7-). Overall, the analysis of the C+V+ and C-V+ cohort independent from each other, revealed cross-reactive T_CM,_ T_NAIVE,_ T_EM,_ T_EMRA_ CD4+ and CD8+ memory subsets, recognizing equally all tested peptides (**Figures S6, S7**). Among the C+V+ cohort we documented a clear predominance of the T_CM_ CD4+ cells against all tested VOC (**Figure S8**) and significantly higher frequencies of A T_EM_ CD8+ among the rest CD8+ memory subsets (**Figure S9**) (Kruskal-Wallis test and Mann-Whitney Test). The C-V+ memory CD4+ and CD8+ T cell subsets were equally represented (**Figure S8**), except for the O T_CM_ CD4+ cells which showed significantly higher frequencies compared to T_EM_ CD4+ cells (**Figure S8D**) (Mann-Whitney Test).

The direct comparison of the memory subsets of C+V+ and C-V+ individuals revealed similar memory T cell frequencies (**Figure S8 and S9**), except for significantly higher WT T_EM_ CD4+ frequencies among the C-V+ cohort compared to the C+V+ (**Figure S8A**).

## 4 Discussion

This study offers a comprehensive analysis of the SARS-CoV-2 specific immunity and cross-recognition potential against the most influential VOC among vaccinated convalescent, uninfected vaccinated and unvaccinated convalescent individuals. Despite the apparent loss of neutralization potential due to viral evolution, a preserved SARS-CoV-2 reactive T cell response with a robust potential for cross-recognition of the alpha, delta and omicron VOC was observed in all 3 studied cohorts. Here, our results are in line with recent studies (13–19, 24) examining the cross-recognition potential of the SARS-CoV-2 memory immune response of unvaccinated convalescent or/and uninfected vaccinated individuals, and expand furthermore our knowledge regarding current SARS-CoV-2 immune dynamics.

We demonstrate that unifected vaccinated adults after 3^rd^ vaccination generate superior multifaceted humoral SARS-CoV-2 memory against VOC compared to unvaccinated convalescent adults. However, combined SARS-CoV-2 vaccination and infection induces more robust humoral response. This comes in agreement with independent studies, underlying that SARS-CoV-2 infection and vaccination together lead to improved protection from reinfection and disease (25, 26).

Despite the expected strongly differentiated NAb titers (3), it is of interest to observe comparable frequencies of SARS-CoV-2 memory T cells across the studied cohorts. Other studies (27, 28) have also observed that the S-specific T cell immunity does not seem to be boosted after vaccination. However, emerging data indicate that SARS-CoV-2 vaccination diversifies the CD4+ S-reactive T cell repertoire in patients with prior SARS-CoV-2 infection (29, 30) or vaccine breakthrough infection with omicron (31). Furthermore, according to Röltgen et al. and Suryawanshi et al vaccination generates antibody breadth with the ability to bind VOC, whereas SARS-CoV-2 infection sustains immune imprinting due to the loss of germinal centers in COVID-19 (31–34).

A key motivation for our study was to elucidate the immunological response in current vaccination strategies while anticipating for next generation vaccines, as the first data of preclinical and clinical trials with previously primed subjects, receiving booster vaccination with updated versions of the currently used mRNA vaccines are not expected. Inoculation with omicron S-mRNA booster does not seem to offer a significant advantage compared to the existing WT S-mRNA vaccines (35–38). However booster vaccination with a monovalent WT or beta, or bivalent WT + beta formulation significantly boosts the pre-existing neutralizing antibodies against the parental WT strain (35, 39). Notably, Ying et al. observed, that primary vaccination series with mRNA-1273.529 potently neutralized B.1.1.529 but showed limited inhibition of historical or other SARS-CoV-2 variants (37). Therefore second generation vaccines opting for the S sequence that induces the breadest VOC-neutralizing response and not for the most concerning variant at any given time may be a wiser strategy (26).

There are some limitations of this study that should be addressed. We analysed the immunogenicity but not the protective capacity of the applied vaccination strategies. The C-V+ cohort included significantly younger participants compared to the C+V+ study group. In addition the sample size of the C+V- and C-V+ cohorts was limited. Further studies exploring the role of preexisting humoral and cellular immunity upon acute reinfection or vaccine breakthrough infection are for this purpose needed.

The question arises whether widespread delta and omicron vaccine breakthrough infections or reinfections could accelerate the end of the pandemic. The superiority of SARS-CoV-2 humoral response among vaccinated convalescent adults in combination with the preserved cellular immunity may accelerate viral elimination and the resolvement of symptomatic COVID-19 disease in case of vaccine breakthough infection or reinfection. This phenomenon could explain the mildly symptomatic SARS-CoV-2 infections with repetive molecular SARS-CoV-2 negativity, despite the strong clinical/history evidence for SARS-COV-2 infection. Next generation-vaccines with broader antigenic spectrum are urgently needed with the potential to induce long lasting neutralizing antibodies-ideally sterilizing immunity, in order to secure public´s compliance with future vaccination campaigns.

## Data availability statement

The raw data supporting the conclusions of this article will be made available by the authors, without undue reservation.

## Ethics statement

The studies involving human participants were reviewed and approved by Ethics Committee of University Hospital Essen (20-9753-BO). The patients/participants provided their written informed consent to participate in this study.

## Author contributions

KP, NB, MA and US participated in research design. KP, MK, MZ, JJ and SD participated in data curation and sample acquisition. KP and NB participated in the writing of the paper. NB, OW, TW and US participated in funding acquisition and project administration. KP, MA, TM, SP and AK participated in the performance of the research. NB, TW, OW and US contributed new reagents or analytic tools. KP, NB and MA participated in data analysis. All authors contributed to the article and approved the submitted version.

## Funding

This work was supported by grants of Mercator Foundation, EFRE grant for COVID.DataNet. NRW, AiF grant for EpiCov, and BMBF for NoChro (FKZ 13GW0338B).

## Conflict of interest

The authors declare that the research was conducted in the absence of any commercial or financial relationships that could be construed as a potential conflict of interest.

## Publisher’s note

All claims expressed in this article are solely those of the authors and do not necessarily represent those of their affiliated organizations, or those of the publisher, the editors and the reviewers. Any product that may be evaluated in this article, or claim that may be made by its manufacturer, is not guaranteed or endorsed by the publisher.
