# Supplementary material for: Superior humoral immunity in vaccinated SARS-CoV-2 convalescence as compared to SARS-COV-2 infection or vaccination
Source: Front Immunol. 2022 Oct 31;13:1031254. doi: 10.3389/fimmu.2022.1031254 (PMC9659602; doi:10.3389/fimmu.2022.1031254)
Supplement: Supplementary file 1 [file DataSheet_1.pdf]

**Superior humoral immunity in vaccinated SARS-CoV-2 convalescence as compared to SARS-COV-2 infection or vaccination**

## Supplemental Table of Contents

### Supplementary Tables

**Table S1:** Fluorochrome coupled antibodies and fluorescent dye for analysis of SARS-CoV-2 reactive T cells

### Supplementary Figures

**Figure S1.** Flow cytometry gating strategy for identification and quantification of SARS-CoV-2 reactive T cells
**Figure S2.** Steady decline of Nabs titers along the VOCs evolvement with the lowest neutralization capacity for omicron VOC
**Figure S3.** SARS-CoV-2 reactive CD4+ and CD8+ T cells cross-recognize VOC after infection and/or vaccination
**Figure S4.** Preservation of functional activity in CD4+ T-cells directed against different VOC
**Figure S5.** Preservation of functional activity in CD8+ T-cells directed against different VOC
**Figure S6.** Preserved cross-reactive C+V+ and C-V+ CD4+ T cell memory
**Figure S7.** Preserved cross-reactive C+V+ and C-V+ CD8+ T cell memory
**Figure S8.** Predominance of the $T_{CM}$ CD4+ cell memory among the C+V+
**Figure S9.** Comparable SARS-CoV-2 reactive CD8+ T cell memory subsets among C+V+ and C-V+

**Table S1:** Fluorochrome coupled antibodies and fluorescent dye for analysis of SARS-CoV-2 reactive T cells

| Antibodies or fluorescent dye | Fluorochrome | Source | Cat. Nr. |
|---|---|---|---|
| Fixable Viability-Dye | eFluor780 | eBioscience | 65-0865-14 |
| anti CCR7 (clone G043H7) | PerCP-Cy5.5 | BioLegend | 353220 |
| anti CD4 (clone OKT4) | A700 | BioLegend | 317426 |
| anti CD8 (clone RPA-T8) | V500 | BD Biosciences | 560775 |
| anti CD45RA (clone HI100) | BV605 | BioLegend | 304134 |
| anti Granzyme B (clone GB11) | FITC | BioLegend | 515403 |
| anti IL2 (clone MQ1-17H12) | PE | BioLegend | 500307 |
| anti CD185(CXCR5) (clone MP4-25D2) | PE-Dazzle594 | BioLegend | 356927 |
| anti CD137 (4-1BB) (clone 4B4-1) | PE-Cy7 | BioLegend | 309818 |
| anti CD154 (CD40L) (clone 24-31) | A647 | BioLegend | 310818 |
| anti TNFα (clone MAb11) | eFluor450 | eBioscience | 48-7349-42 |
| anti IFNγ (clone 4S.B3) | BV650 | BioLegend | 502538 |
| anti CD3 (clone OKT3) | BV785 | BioLegend | 317330 |

## Supplementary figures

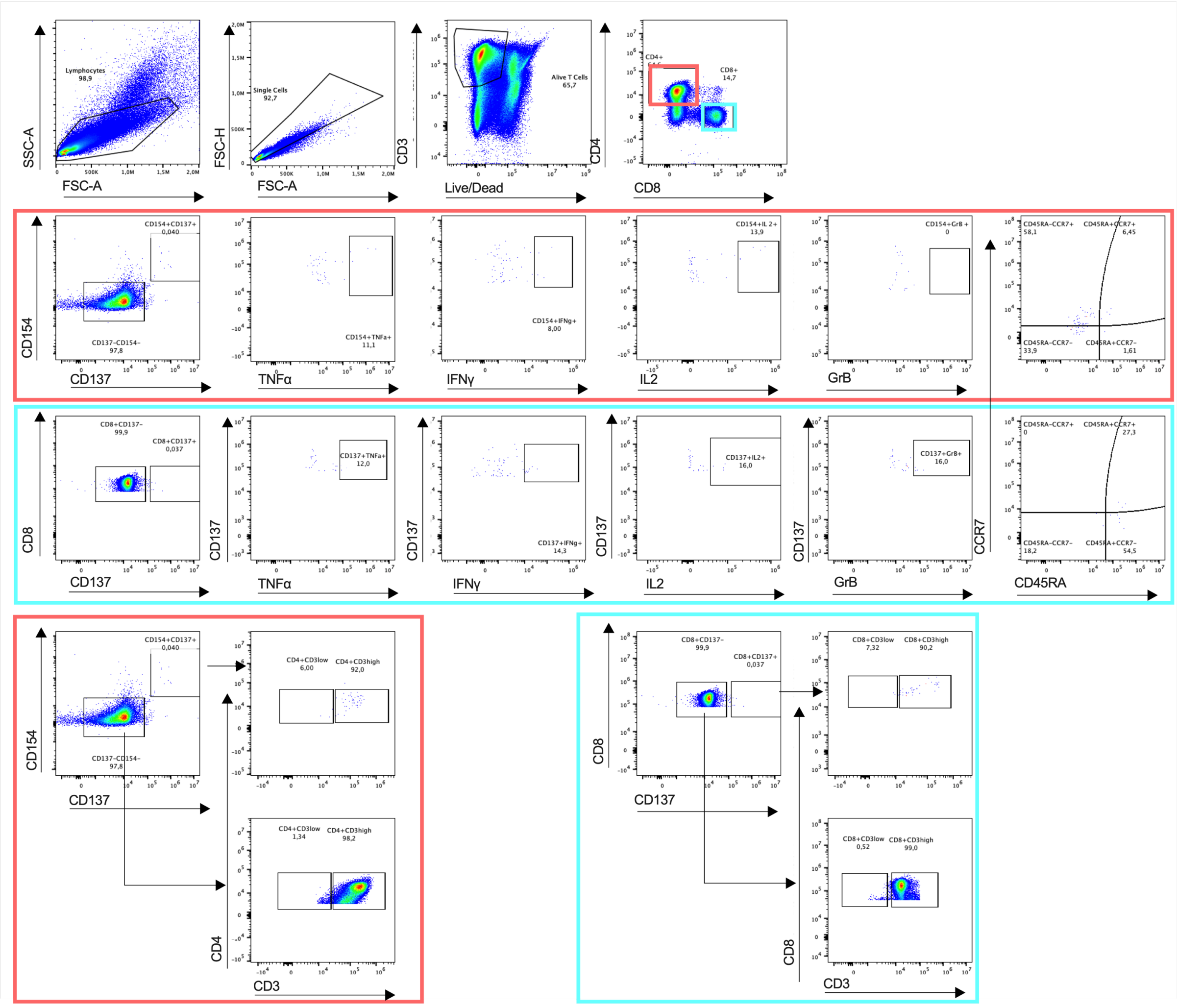


**Figure S1. Flow cytometry gating strategy for identification and quantification of SARS-CoV-2 reactive T cells.** PBMCs were stimulated for 16 h with one of the following: the pool of B1.617.2 (delta) Spike mutant peptides (Miltenyi Biotec), their reference pool of peptides (Miltenyi Biotec), the pool of B.1.1529 (omicron) Spike mutant peptides (Miltenyi Biotec), their reference pool of peptides (Miltenyi Biotec), the complete sequence WT S-protein (Miltenyi Biotec) or left untreated as a control. Living single lymphocytes were analyzed for expression of CD3, CD4, and CD8. CD4+ T cells (orange boxes) were analyzed for the expression of CD154 and CD137. CD8+ T cells (blue boxes) were analyzed for expression of CD137. Both CD4+ and CD8+ T cells were further analyzed for the production of cytokines IFNγ, TNFα, IL2 and GrB. Evaluation of the memory subsets was performed using the markers CCR7 and CD45RA ($T_{CM}$=CD45RA-CCR7+, $T_{NAIVE}$=CD45RA+CCR7+, $T_{EM}$=CD45RA-CCR7- $T_{EMRA}$=CD45RA+CCR7-). Furthermore, CD4+CD154+CD137+, CD8+CD137+ and cells were analyzed for the expression of CD3low. Representative example of 42 C+V+, 8 C-V+ and 7 C+V- study subjects. Plots of a C+V+ study subject are depicted.

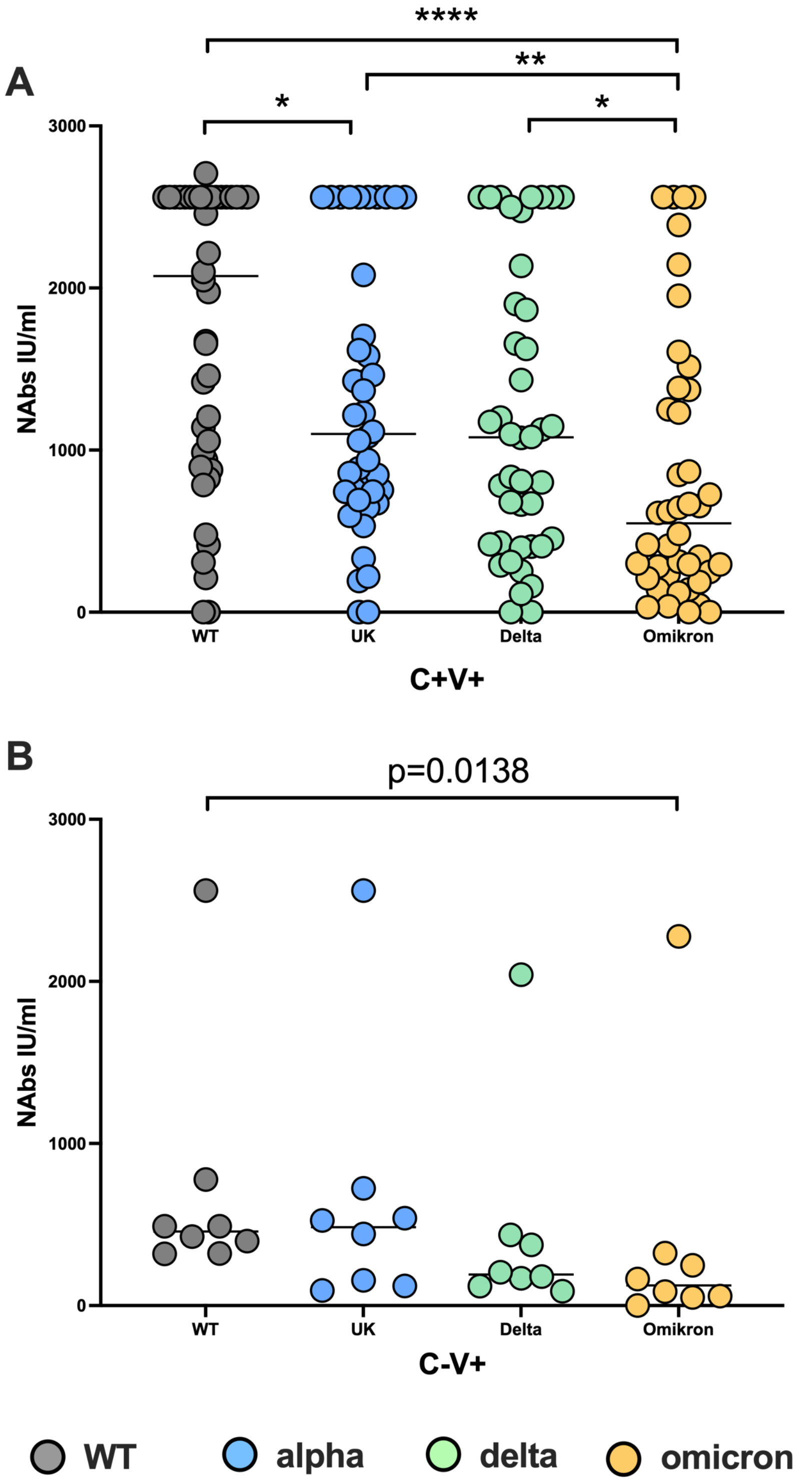


**Figure S2. Steady decline of Nabs titers along the VOCs evolvement with the lowest netrailzation capacity for omicron VOC.** Analysis of WT, alpha, delta and omicron NAbs titers via pseudovirus neutralization assay. A) NAbs against VOC among C+V+. B) NAbs against VOC among C-V+. Scatterplots show line at median. Unpaired data were compared

with Mann-Whitney-test. $P<0.05$ was considered significant, only significant p values are documented in the figures.

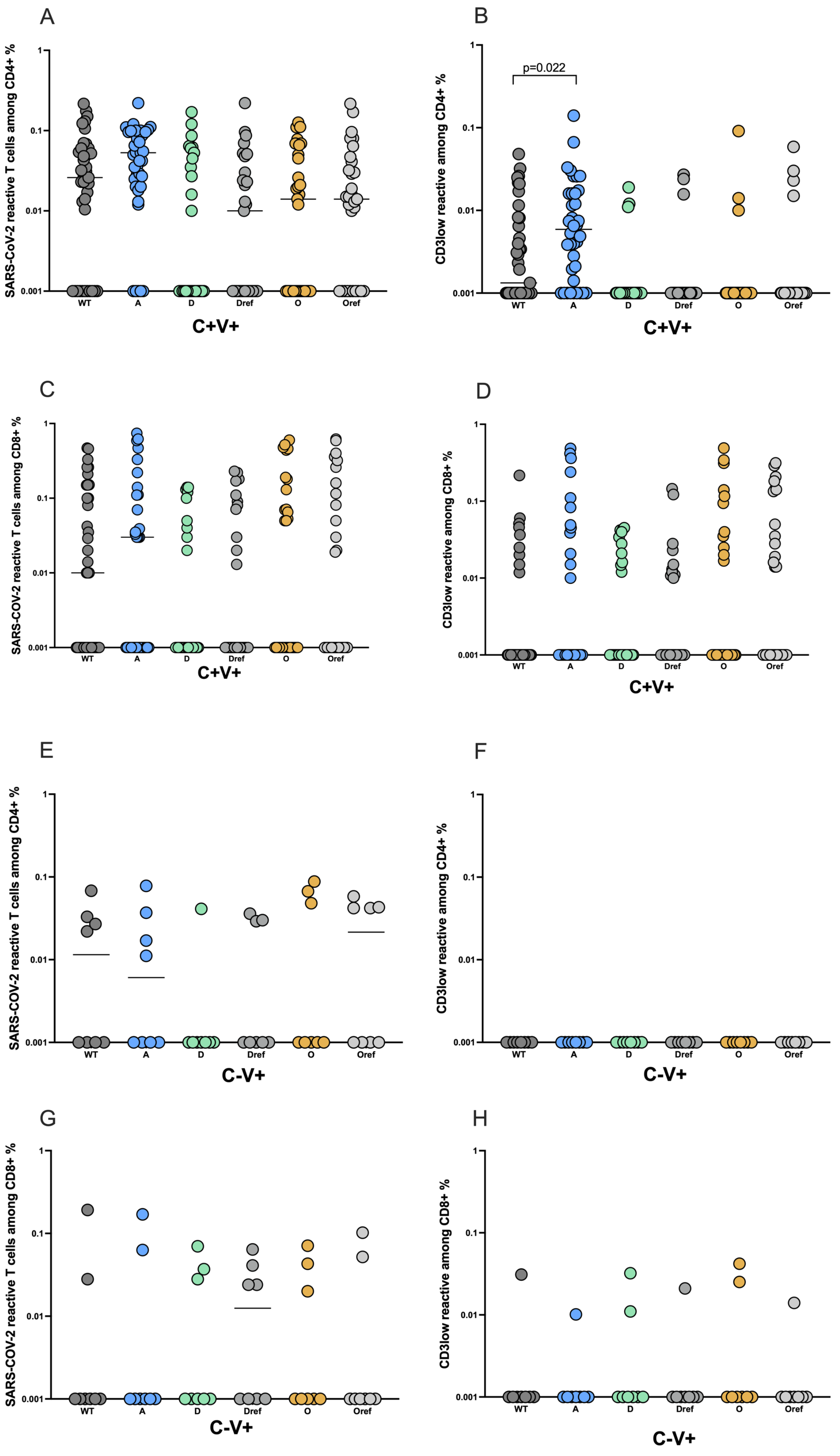
A
SARS-CoV-2 reactive T cells among CD4+ %
1
0.1
0.01
0.001
WT
A
D
Dref
O
Oref
C+V+
B
CD3low reactive among CD4+ %
p=0.022
C+V+
C
SARS-COV-2 reactive T cells among CD8+ %
C+V+
D
CD3low reactive among CD8+ %
C+V+
E
SARS-COV-2 reactive T cells among CD4+ %
C-V+
F
CD3low reactive among CD4+ %
C-V+
G
SARS-COV-2 reactive T cells among CD8+ %
C-V+
H
CD3low reactive among CD8+ %
C-V+

**Figure S3. SARS-CoV-2 reactive CD4+ and CD8+ T cells cross-recognize VOC after infection and/or vaccination.** Characterization of SARS-CoV-2 S-reactive T cells in C+V+ and C-V+ study subjects. Blood samples of the study subjects were stimulated with SARS-CoV-2 peptides and analyzed by flow cytometry. SARS-CoV-2 S-reactive CD4+ and CD8+ T cells are defined as CD4+CD154+CD137+ and CD8+CD137+ cells respectively. (A) Frequencies of WT- or ref-, alpha-, delta- and omicron-reactive CD4+ T cells among C+V+. (B) Avidity of SARS-CoV-2 reactive CD4+ T cells was approached by determining the CD3low+ cells among CD4+CD154+CD137+ cells. Frequencies of WT- or ref-, alpha-, delta- and omicron-reactive CD4+CD3low+ T cells among C+V+. (C) Frequencies of WT- or ref-, alpha-, delta- and omicron-reactive CD8+ T cells among C+V+. (D) Avidity of SARS-CoV-2 reactive CD8+ T cells was approached by determining the CD3low+ cells among CD8+CD137+ cells. Frequencies of WT- or ref-, alpha-, delta- and omicron-reactive CD8+CD3low+ T cells among C+V+. (E) Frequencies of WT- or ref-, alpha-, delta- and omicron-reactive CD4+ T cells among C-V+. (F) Frequencies of WT- or ref-, alpha-, delta- and omicron-reactive CD4+CD3low+ T cells among C-V+. (G) Frequencies of WT- or ref-, alpha-, delta- and omicron -reactive CD8+ T cells among C-V+. (H) Frequencies of WT- or ref-, alpha-, delta- and omicron-reactive CD8+CD3low+ T cells among C-V+. Antigen-reactive responses were considered positive after the non-reactive background was subtracted, and more than 0.01% were detectable. Scatterplots show line at median. Unpaired data were compared with Mann-Whitney-test. $P<0.05$ was considered significant, only significant p values are documented in the figures.

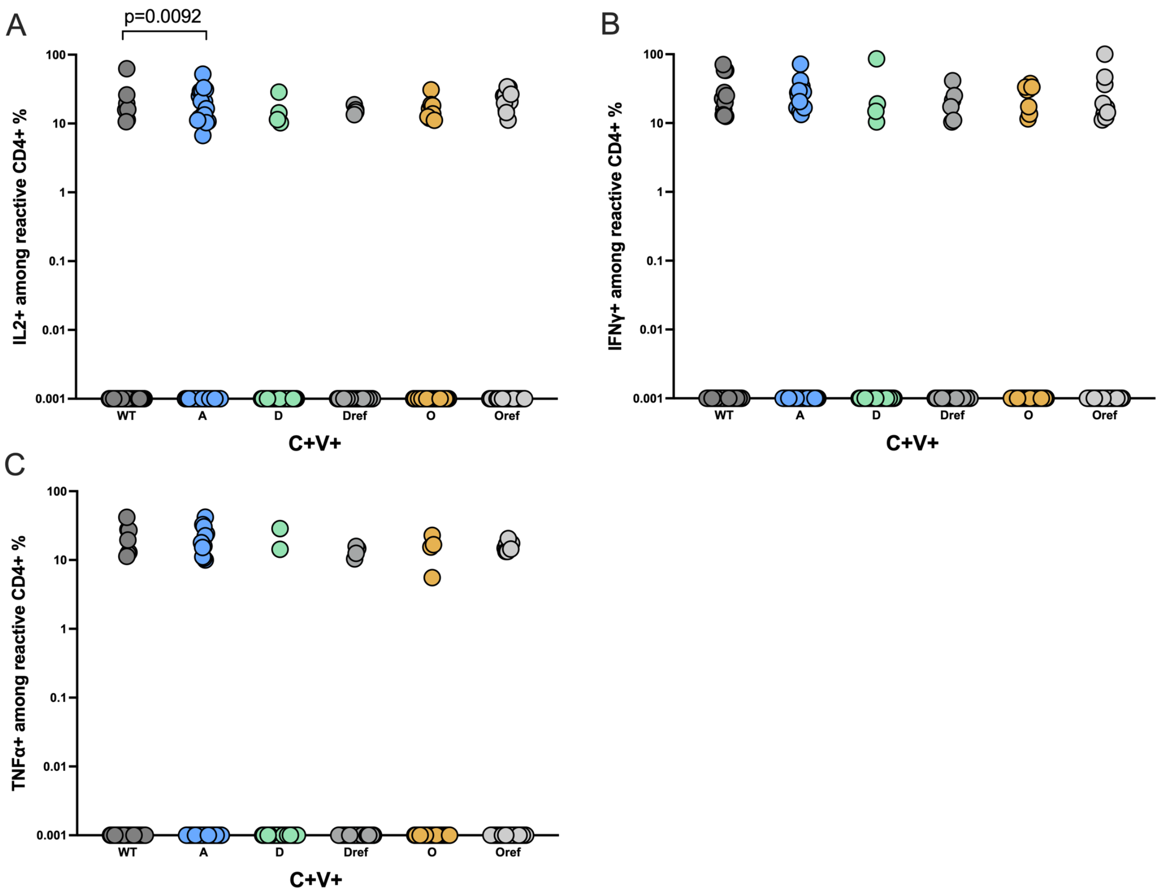


**Figure S4. Preservation of functional activity in CD4+ T-cells directed against different VOC.** SARS-CoV-2-reactive CD4+ cells of C+V+ subjects were analyzed for GrB, IFNγ, IL2 or TNFα production. A) IL2+. B) IFNγ+ and C) TNFα+.

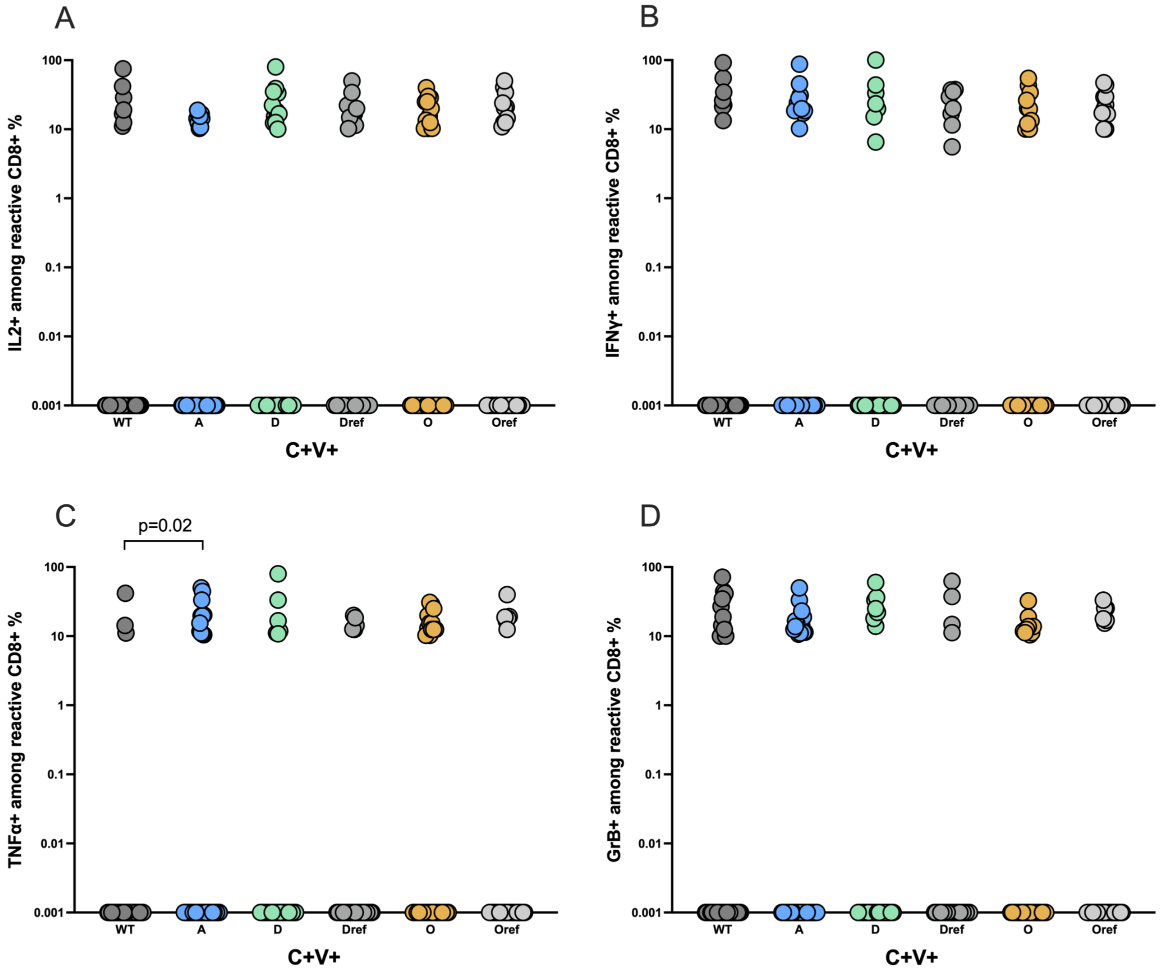


**Figure S5. Preservation of functional activity in CD8+ T-cells directed against different VOC.** SARS-CoV-2-reactive CD8+ cells of C+V+ subjects were analyzed for GrB, IFNγ, IL2 or TNFα production. A) IL2+, B) IFNγ+, C) TNFα+ and GrB+.

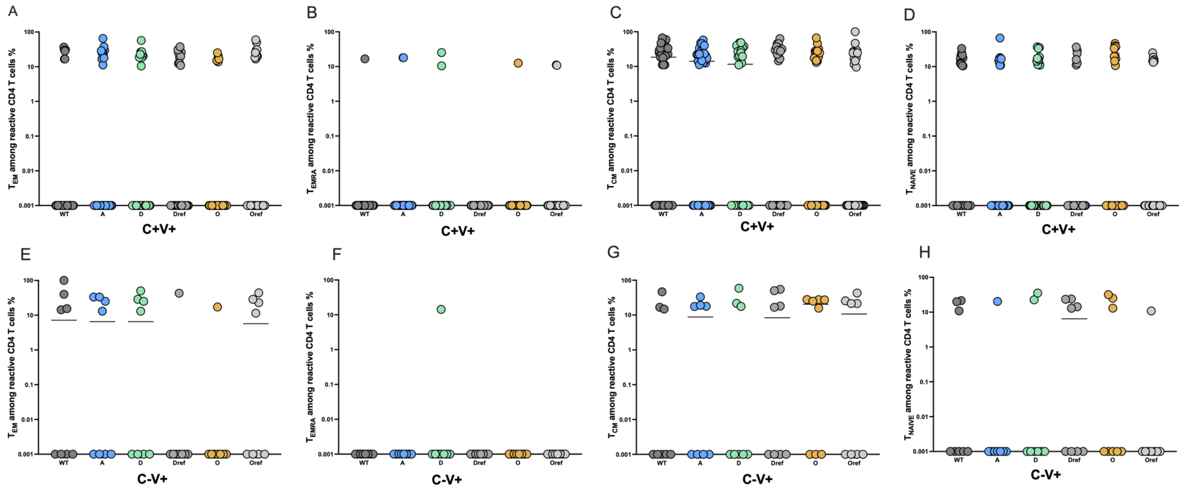

**Figure S6. Preserved cross-reactive C+V+ and C-V+ CD4+ T cell memory.** Evaluation of the memory subsets was performed using the markers CCR7 and CD45RA (TCM=CD45RA-CCR7+, TNAIVE=CD45RA+CCR7+, TEM=CD45RA-CCR7- TEMRA=CD45RA+CCR7-). Frequencies of the memory subsets of WT- reactive CD4+ of C+V+ and C-V+ study groups. (A) Frequencies of WT- or ref-, alpha, delta and omicron-reactive CD4+ $T_{EM}$ cells among C+V+. B) Frequencies of WT- or ref-, alpha, delta and omicron-reactive CD4+ $T_{EMRA}$ cells among C+V+. C) Frequencies of WT or ref, alpha, delta and omicron-reactive CD4+ $T_{CM}$ cells among C+V+. D) Frequencies of WT- or ref-, alpha, delta and omicron-reactive CD4+ $T_{NAIVE}$ cells among C+V+. E) Frequencies of WT- or ref-, alpha, delta and omicron-reactive CD4+ $T_{EM}$ cells among C-V+. F) Frequencies of WT- or ref-, alpha, delta and omicron-reactive CD4+ $T_{EMRA}$ cells among C-V+. G) Frequencies of WT- or ref-, alpha, delta and omicron-reactive CD4+ $T_{CM}$ cells among C-V+. H) Frequencies of WT- or ref-, alpha, delta and omicron-reactive CD4+ $T_{NAIVE}$ cells among C-V+.

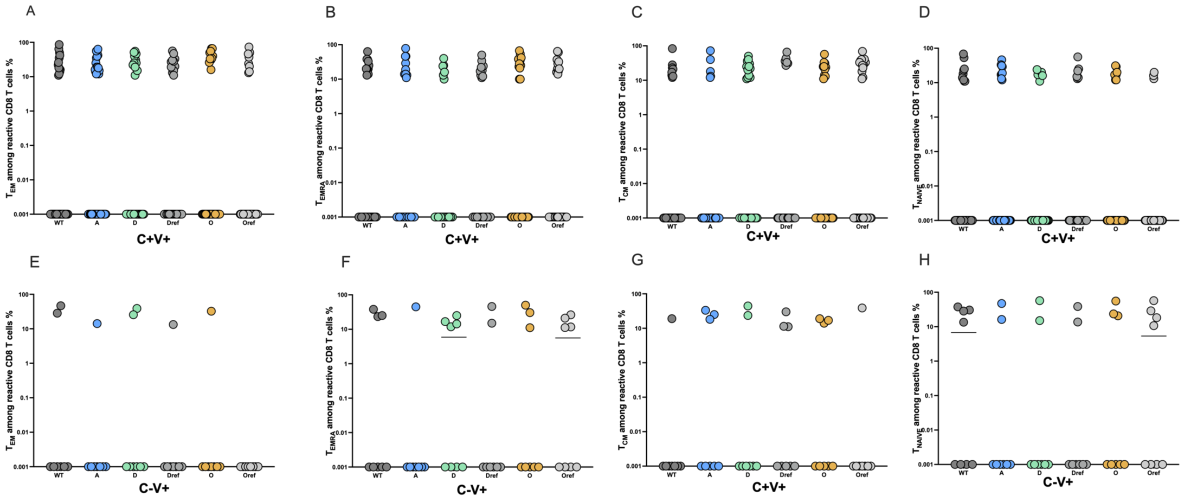


**Figure S7. Preserved cross-reactive C+V+ and C-V+ CD8+ T cell memory.** Evaluation of the memory subsets was performed using the markers CCR7 and CD45RA (TCM=CD45RA-CCR7+, TNAIVE=CD45RA+CCR7+, TEM=CD45RA-CCR7- TEMRA=CD45RA+CCR7-). Frequencies of the memory subsets of WT- reactive CD4+ of C+V+ and C-V+ study groups. (A) Frequencies of WT- or ref-, alpha, delta and omicron-reactive CD8+ $T_{EM}$ cells among C+V+. B) Frequencies of WT- or ref-, alpha, delta and omicron-reactive CD8+ $T_{EMRA}$ cells among C+V+. C) Frequencies of WT- or ref-, alpha, delta and omicron-reactive CD8+ $T_{CM}$ cells among C+V+. D) Frequencies of WT- or ref-, alpha, delta and omicron-reactive CD8+ $T_{NAIVE}$ cells among C+V+. E) Frequencies of WT- or ref-, alpha, delta and omicron-reactive CD8+ $T_{EM}$ cells among C-V+. F) Frequencies of WT- or ref-, alpha, delta and omicron-reactive

CD8+ $T_{EMRA}$ cells among C-V+. G) Frequencies of WT- or ref-, alpha, delta and omicron-reactive CD8+ $T_{CM}$ cells among C-V+. H) Frequencies of WT- or ref-, alpha, delta and omicron-reactive CD8+ $T_{NAIVE}$ cells among C-V+.

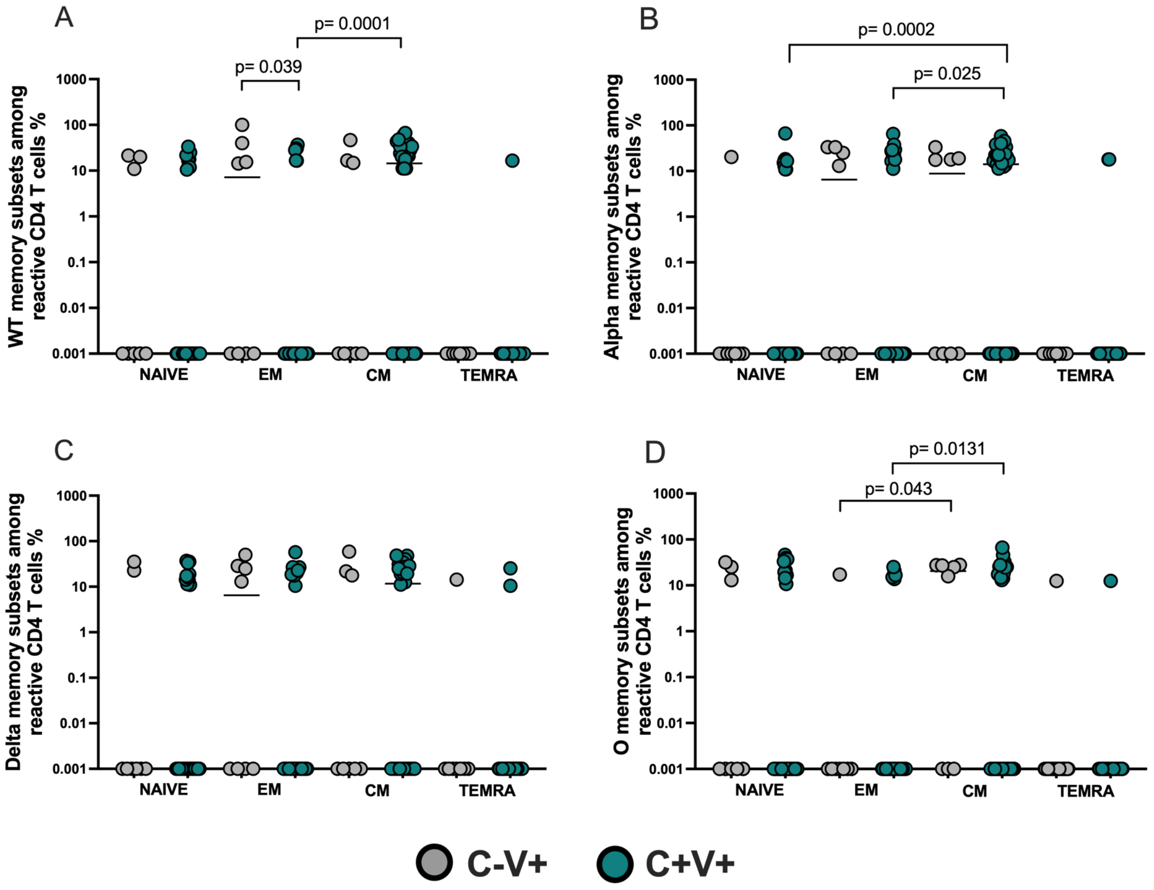


**Figure S8. Predominance of the $T_{CM}$ CD4+ cell memory among the C+V+.** Comparison of SARS-CoV-2 S-reactive T cell memory subsets among C+V+ and C-V+ study subjects. A) Frequencies of WT-reactive CD4+ T cell memory subsets. (B) Frequencies of alpha-reactive CD4+ T cell memory subsets. C) Frequencies of delta-reactive CD4+ T cell memory subsets. D) Frequencies of omicron-reactive CD4+ T cell memory subsets.

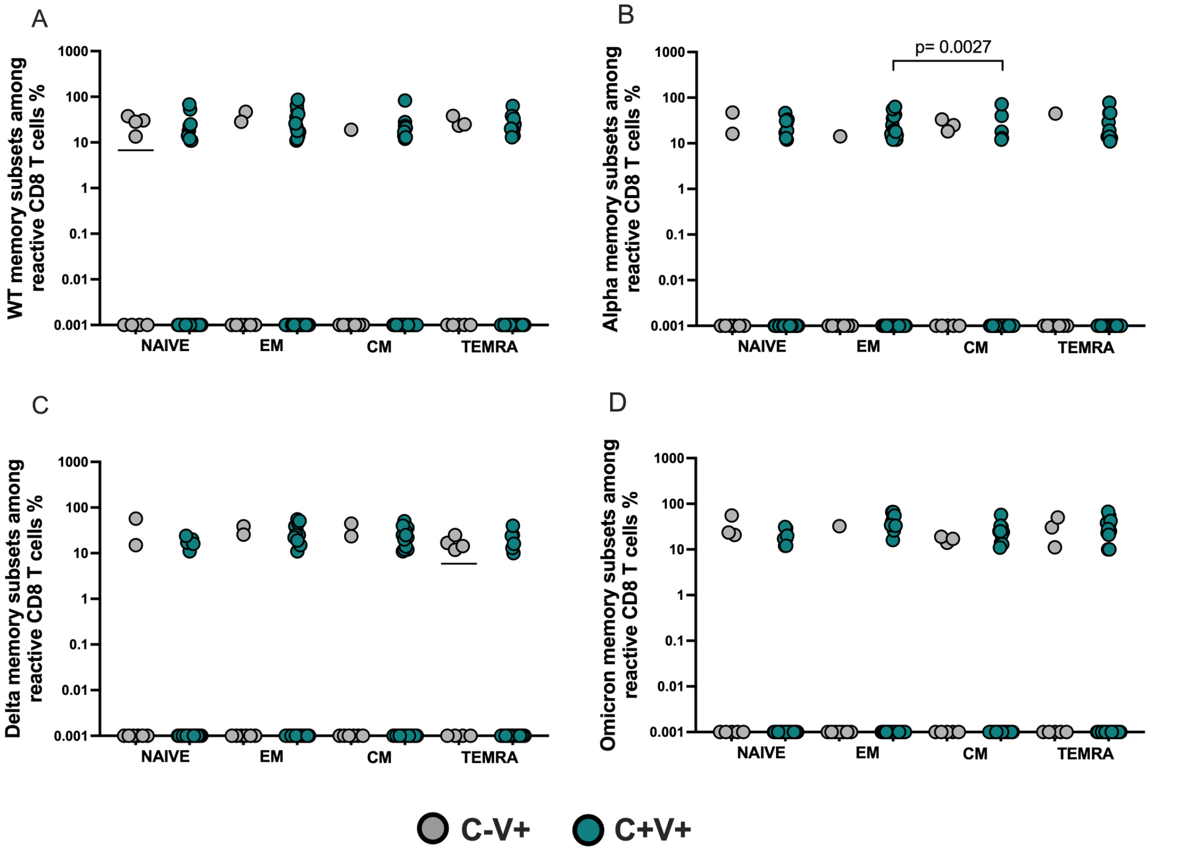


**Figure S9. Comparable SARS-CoV-2 reactive CD8+ T cell memory subsets among C+V+ and C-V+.** A) Frequencies of WT-reactive CD8+ T cell memory subsets. (B) Frequencies of alpha-reactive CD8+ T cell memory subsets. C) Frequencies of delta-reactive CD8+ T cell memory subsets. D) Frequencies of omicron-reactive CD8+ T cell memory subsets.
